# Editorial: Inborn Errors of Immunity (IEI) breaking immune homeostasis and tolerance: a key role for T regulatory cells

**DOI:** 10.3389/fimmu.2024.1532079

**Published:** 2024-12-19

**Authors:** Veronica De Rosa, Georgios Sogkas, Rosa Bacchetta

**Affiliations:** ^1^ Institute for Endocrinology and Experimental Oncology Gaetano Salvatore, Department of Biomedical Sciences, National Research Council (CNR), Naples, Italy; ^2^ Department of Rheumatology and Immunology, Hannover Medical School, Resolving Infection Susceptibility (RESIST) - Cluster of Excellence 2155, Hannover Medical School, Hannover, Germany; ^3^ Department of Pediatrics, Division of Hematology, Oncology, Stem Cell Transplantation and Regenerative Medicine, Stanford University School of Medicine, Stanford, CA, United States

**Keywords:** inborn error immunity, primary immumunodeficiencies, primary immune disorders, tregopathy, autoimmunity, epigenetics

An emerging area of research in immunology focuses on the molecular mechanisms underlying “Inborn Errors of Immunity” (IEI). IEI are typically caused by mutations in genes that regulate various processes, such as immune cell development, differentiation and signaling (1). The discovery of the genetic background has been pivotal in understanding why certain individuals are more susceptible to infections and immune-related complications. Notably, while some patients with IEI present with clinical symptoms in early childhood, others manifest later in life, underscoring the broad impact of these conditions across the lifespan. The latest update of the International Union of Immunological Societies (IUIS) classifies IEI into the following 9 groups: 1. Combined immunodeficiencies. 2. Combined immunodeficiencies with syndromic characteristics. 3. Predominant antibody deficiencies. 4. Diseases of immune dysregulation. 5. Congenital phagocyte defects. 6. Defects in the intrinsic and innate immunity. 7. Autoinflammatory diseases. 8. Complement deficiencies. 9. Bone marrow failure. Disease in some of aforementioned groups is dominated by infections, while others manifest primarily with variable form of immune dysregulation. In this complex scenario, disorders presenting with a phenotype caused by loss of immune tolerance leading to autoimmunity, autoinflammation, lymphoproliferation, and/or severe atopy have come to be recognized as Primary Immune Regulatory Disorders (PIRDs). They encompass a distinct set of disorders secondary to failure in different immune regulatory pathways; hence, this enables a subgrouping into different categories. In 2018, the term Tregopathies was first introduced: it refers to a group of IEI in which the affected target cells are the regulatory T cells (Treg). This group initially included mutations in *FOXP3*, *CD25*, *CTLA-4*, *LRBA*, *BACH2*, *IL10*, and gain of function (GOF) of *STAT3*. Since then, the IUIS expert committee has added new genes to this category, including mutations in *FERMT1*, *CD122*, *DEF6*, and *IKAROS* GOF (2, 3). In this Research Topic, Kennedy-Batalla et al. provide a comprehensive overview of Treg in IEI by focusing on: i) advances and controversies in the evaluation of Tregs; ii) current knowledge and gaps of Treg disturbances in Tregopathies and other IEI, and iii) potential of Treg cell-based therapies for IEI with immune dysregulation. This non-systematic targeted literature review aimed at summarizing both diagnostic and therapeutic approaches to IEI associated with Treg dysfunction. Since Tregs, particularly those committed in the thymus (tTregs), play a non-redundant role in the control of autoimmune and inflammatory diseases, it is critical to identify the relevant network of epigenomic interactions governing tTregs and their possible alterations in IEI. Raposo et al. used the genome-wide expression (RNA-seq) and chromatin accessibility maps (ATAC-seq) of purified CD4 single-positive (CD4SP) Tregs and conventional T cells (Tconv) from human thymuses to define their expression signature and quantify genome-wide transcription factor (TF) binding. Applying an artificial intelligence approach, they uncovered the main Gene Regulatory Modules (GRM) shaping the identity of tTregs in the human thymus. The identified genes are those playing a key role in the regulation of autoimmune and inflammatory processes. They also explored the GRMs of human thymic Tregs to decipher complex immune disorders, by analyzing mutational hotspots in a cohort of patients with Common Variable Immunodeficiency (CVID), the most frequent symptomatic Primary Immunodeficiency (PID) featuring severe autoimmune and inflammatory manifestations, not yet associated with monogenic mutations. CVID patients are characterized by decreased class-switched memory B cells (4) but autoimmune manifestations - that are associated with low Treg levels - can also occur (5). The study results demonstrated - for the large majority of GRM genes - a higher prevalence of mutations in the CVID cohort (Raposo et al.). Tregs have been also characterized in Predominantly Antibody Deficiencies (PADs), a group of IEI with poor antibody responses, increased susceptibility to infections and chronic inflammatory disorders (6). Immunoglobulin G subclass deficiency (IgGsd) is a mild form of PAD characterized by increased frequencies of infections, and reduced levels of at least one IgG subclass. Most patients with IgGsd had one or several comorbidities such as autoimmunity, atopy, or reduced lung function. Interestingly, Wågström and colleagues, identified lower levels of activated Tregs in IgGsd patients were observed, partly restored during immunoglobulin replacement therapy (IgGRT), thus suggesting the involvement of Tregs in the pathogenesis and development of PADs (Wågström et al.). Another key aspect in the IEI field concerns regulatory B cells (Bregs), widely accepted as an important modulatory component that suppresses T cell differentiation and promotes peripheral tolerance. Indeed, the breakdown of their activity associated with both autoimmunity and immunodeficiency (7, 8). Although most PIRDs are early-onset diseases, to date there are limited reference values for the regulatory populations (Tregs and Bregs) in the pediatric population, both in terms of phenotypic and functional characterization. In this Research Topic, Luo et al. describe the changes in Tregs and Bregs observed in a healthy pediatric population showing that they are abundant before the age of 7 and 3, respectively; this evidence reinforces the concept that the immunotolerance process mainly occurs during early childhood. Pediatric PID population requires more detailed assessment of the immune status because autoimmunity often develops at an early age. Intriguingly, pediatric PID patients having IgG deficiency paradoxically display autoreactive antibodies (i.e., anti-tTG, anti-DGP, anti-SS-B, anti-Sm) that associate with autoimmune disorders, typically celiac disease (CD) (9). Hence, measurement of autoantibodies might be a useful screening test for autoimmune diseases in PID patients. The association of CD with PIDs (i.e. sIgAD and CVID) suggests shared pathomechanisms for immunodeficiency and autoimmunity. Indeed, the process leading to the development of autoimmunity in PIDs is complex and includes dysregulated B cell differentiation and germ‐center reactions together with altered T cell tolerance and uncontrolled lymphocyte proliferation and differentiation (10). Scarmozzino et al. provide a comprehensive view of refractory celiac diseases (RCD-I and -II) and enteropathy-associated T-cell lymphoma (EATL), two rare, yet severe, complications of CD. RCD-I and RCD-II are distinct disorders stemming from a disease initially driven by abnormal T-cell immune responses against gluten-derived peptides in genetically susceptible individuals (11). More in detail, RCD-I represents a gluten-independent dysimmune reaction of the small bowel, while RCD-II can be regarded as an aggressive *in situ* T cell lymphoma with high risk of EATL progression. The authors addressed the biology and clinical-pathological features of such conditions, highlighting unique disease patterns and recurrent genetic events. Disruptions in the epigenome are increasingly being recognized as a common pathogenic mechanism underlying rare IEI (12). This is the case of Immunodeficiency, Centromeric instability and Facial anomalies (ICF) syndrome, a rare autosomal genetic disorder characterized by hypogammaglobulinemia and chromosomal instability accompanied by DNA hypomethylation (13). ICF patients have naïve but lack memory B-cells and plasma cells. Since some ICF patients suffer from recurrent viral and/or fungal infections, ICF patients have been suspected of having concomitant T-cell immunodeficiency. While less common, a subgroup of patients also exhibits T-cell abnormalities alongside B-cell anomalies, including reduced regulatory T-cells and increased effector memory T- and follicular helper T-cells. Their pathological variant genes and the complex immunophenotype and have been accurately recapitulated in a brief review from Unoki, which explored the intersection among epigenetics, DNA repair, and immunology. The dysregulation of immunoglobulin signaling in B-cells, the imbalance in T-cell subsets, and/or satellite RNA-mediated activation of innate immune response potentially explain autoimmune manifestations in a subset of patients. However, the understanding of the molecular pathogenesis underlying immune dysregulation in these patients is still in its initial stages. In this Research Topic, Lullo et al. provide a novel human model for ICF-2 subtype 2 (ICF-2), due to pathogenic variants of the *ZBTB24* gene. ZBTB24 is a transcription factor belonging to the Zinc-finger and BTB domain-containing protein family, key regulators of developmental processes. The functional link between *ZBTB24* deficiency and DNA methylation errors is still elusive. By deriving induced pluripotent stem cells (iPSCs) from peripheral CD34^+^-blood cells of a patient, they generated a highly relevant model to explore the role of ZBTB24 in DNA methylation homeostasis, providing a tool to investigate the early molecular events linking ZBTB24 deficiency to the ICF2 clinical phenotype (Lullo et al.). This is particularly relevant to dissecting the complex relationship between the genetic causative mutations and the molecular and clinical phenotypes in human chromatin disorders.

Overall, this Research Topic provides novel evidence to increase our understanding of the function of the immune system but also pave the way for new interventions to improve the lives of individuals affected by IEI and perhaps other related – more common – immune related diseases.
